# Neuroblastoma GD2 Expression and Computational Analysis of Aptamer-Based Bioaffinity Targeting

**DOI:** 10.3390/ijms22169101

**Published:** 2021-08-23

**Authors:** Godfred O. Sabbih, Michael K. Danquah

**Affiliations:** Department of Chemical Engineering, University of Tennessee, Chattanooga, TN 37403, USA; fzp281@mocs.utc.edu

**Keywords:** neuroblastoma, minimal residual disease, tumor biomarker, oncology

## Abstract

Neuroblastoma (NB) is a neuroectodermal embryonic cancer that originates from primordial neural crest cells, and amongst pediatric cancers with high mortality rates. NB is categorized into high-, intermediate-, and low-risk cases. A significant proportion of high-risk patients who achieve remission have a minimal residual disease (MRD) that causes relapse. Whilst there exists a myriad of advanced treatment options for NB, it is still characterized by a high relapse rate, resulting in a reduced chance of survival. Disialoganglioside (GD2) is a lipo-ganglioside containing a fatty acid derivative of sphingosine that is coupled to a monosaccharide and a sialic acid. Amongst pediatric solid tumors, NB tumor cells are known to express GD2; hence, it represents a unique antigen for subclinical NB MRD detection and analysis with implications in determining a response for treatment. This article discusses NB MRD expression and analytical assays for GD2 detection and quantification as well as computational approaches for GD2 characterization based on high-throughput image processing and genomic data analysis.

## 1. Introduction

NB is a solid tumor that originates from the primordial neural crest cells [1]. It is an extracranial tumor of the peripheral sympathetic nervous system that is typically found within the adrenal medulla. It is common amongst children under the age of 15 years, accounts for about 8–10% of all childhood cancers, and has a mortality rate of 15% [1,2]. Depending on the stage, age, histological category, grade of tumor differentiation, status of the MYCN oncogene, chromosome 11q status, and DNA ploidy, NB can be categorized as low-, intermediate-, or high-risk according to the International Neuroblastoma Risk Group (INRG) [3]. High-risk NB (HRNB) accounts for about 50% of cases [4].

Depending on the risk category and prognosis, treatment may include surgery to remove some or all of the localized tumor. Typically, low-risk tumors may be monitored for spontaneous differentiation or regression and, if needed, chemotherapy or radiotherapy may be performed [4]. Current treatment methods used in HRNB maintain a combination of induction chemotherapy, local treatment by surgery and radiotherapy, consolidation with high-dose chemotherapy, and reinjection of autologous stem cell transplantation (ASCT), as well as maintenance therapy in residual disease using anti-GD2 monoclonal antibody (MoAB)-based treatments [5]. Anti-GD2 MoAB is an effective treatment method and employs drugs such as ch14.18 (dinutuximab) in conjunction with granulocyte-macrophage colony-stimulating factor (GM-CSF), interleukin-2 (IL-2), and isotretinoin [6].

It is worth noting that major advancements made towards the treatment of NB relate to the treatment of HRNB cases with an overall chance of survival of 20% at 2 years for patients treated with monoclonal antibody ch14.18 and anti-GD2 antibody plus granulocyte-macrophage colony-stimulating factor (GMCSF) and interleukin-2 [6,7]. Patients with advanced NB have also benefited greatly from myeloablative therapies, which involve the use of high-dose chemotherapy for the destruction of cancerous cells followed by either autologous or allogeneic bone marrow transplantation to replace lost bone marrow and to support the reconstruction of blood and the immune system. Studies have shown that myeloablative therapy in addition to autologous bone marrow transplantation (ABMT) significantly increase event-free-survival (EFS) rates [8,9].

However, relapse continues to persist even after intensive treatment, and this is a direct result of a minimal residual disease (MRD), which constitutes small, drug-resistant tumor cells that persist during treatments. Reported data shows a 5-year post relapse survival rate of only 8%, and 4% for patients diagnosed with metastasis [10]. New and improved early NB MRD detection methods are therefore critical to inform treatment augmentation strategies for HRNB. Presently, the main MRD detection methods applied to NB MRD are reverse transcriptase polymerase chain reaction (RT-PCR) that can detect one tumor cell in about 107 hematopoietic cells (HP), and immunocytological methods that can detect one tumor cell in about 105 HP cells. The former utilizes the chemistry of disease-associated genetic biomolecules while the latter is based on disease-specific antigens. The article focuses on the latter to discuss the enormous potential of one of the NB antigens, GD2, identified as the 12th most specific biomarker of neuroblastoma [11]. Recent studies have shown a reduction in the concentration of circulating GD2 in NB patients in response to therapy, and reappearance in patients who had relapsed, indicating the importance of GD2 as a biomarker of NB [12].

In this article, we discuss some of the characteristics of NB MRD based on the antigen GD2 as an opportunity to develop novel theranostics for NB using bioaffinity approaches such as aptamers. It also discusses computational approaches that can be used for the characterization of NB and other neuroectodermal cancers based on high-throughput image processing of GD2 expression.

## 2. Epidemiology of NB

NB is the most common extracranial solid tumor in infants and children under the age of 15 years; it accounts for about 8–10% of all childhood tumors and is associated with about 15% of all pediatric cancer-related deaths [13]. The incidence rate is about 10.2 cases per million children, with newly reported annual cases of about 500 [13,14]. The median age of diagnosis for NB is about 22 months and almost 90% of cases diagnosed before the age of 5 are predominantly among males [13,15].

Of all the cases of diagnosed NB, only 1–2% are related to genetic causes; most of the cases are possibly related to a myriad of environmental factors, which may include drugs, chemicals, and viruses [15]. In recent years, studies have also shown that familial cases show mutations in the anaplastic lymphoma kinases (ALK) gene located on chromosome 2 and this has catalyzed research into the development of ALK inhibitors as novel therapeutic agents [16,17].

NB survival is also increasing steadily with about 52–74% 5-year survival rates reported [13]. This improvement is largely related to the enhanced cure rates and treatments associated with low-risk NB [1]. Treatment of HRNB remains a challenge. Relapse rates of HRNB have been estimated to be about 50–60% with a very low chance of postrelapse survival [18]. The International Neuroblastoma Risk Group also estimates a median time to relapse of 13.2 months, with 73% of those who relapsed aged 18 months or older. Despite all available treatment modalities, NB patients have low chances of survival as a result of MRD. Early detection of MRD may contribute to enhance the survival rates of NB through treatment augmentations.

## 3. Genomics of Neuroblastoma-GD2 Synthase Gene

NB is genetically heterogeneous and has been shown to develop as a result of several genetic alterations [1,13,15]. An understanding of the underlying genomic characteristics of NB development is essential to assist in the development of new and/or improved therapeutic approaches [19]. A prominent genetic feature of NB that has been the primary focus of diagnosis is MYCN amplification, discovered in 1983 [20]. Its prominence as a biomarker of NB came about after major discoveries that linked its expression to poor prognosis in patients [21]. MYCN amplification alone is, however, not sufficient for stratifying the risk associated with the disease since about 60% of HRNB tumors are without MYCN amplification [22]. Thus, other genetic biomarkers are needed to completely diagnose the malignancy in a higher percentage of patients.

The existence of other genetic aberrations specific to NB has been confirmed with techniques such as comparative genomic hybridization (CGH) [23] and DNA/RNA sequencing [22,23]. CGH, for instance, has been used on NB cell lines to confirm the presence of MYCN amplification as well as a combination of unique chromosomal aberrations: 17q gain and deletions at 1p36, 3p, 4p, 9p, 11q, and 14q regions [24,25]. The expression characteristics of the antigen GD2 is also very crucial in the development of analytical assays for NB. It should be worth noting that the potential of GD2 based theranostic approaches goes beyond NB as there are other neuroectodermal malignancies such as Ewing’s tumor family, osteosarcoma, liposarcoma, rhabdomyosarcoma, retinoblastoma, and fibrosarcoma that also express GD2 [26]. The National Cancer Institute (NCI) rankings for cancer antigens places GD2 as the 12th most important cancer antigen based on a weighted percentage of 9 different criteria based on molecular weights: therapeutic function, immunogenicity, oncogenicity, specificity, expression level and percent of antigen-positive cells, stem cell expression, number of patients with antigen-positive cancers, number of antigenic epitopes, and cellular location of antigen expression [11]. Understanding the genomics and expression characteristics of GD2 would facilitate the development of high-efficacy theranostic technologies with a greater impact on cancer treatments.

GD2 is expressed in some specific cells (neuroectodermal tumors and sarcomas) [27] as a product of the activities of the enzyme GM2/GD2 synthase (1,4-N-acetyl galactosaminyltransferase). GD2 synthase is the main enzyme that catalyzes the conversion of GM3, GD3, and lactosylceramide (LacCer) to GM2, GD2, and asialo-GM2 (GA2) [27]. The gene responsible for the transcription of the GD2 synthase is the GD2 gene, also known as B4GALNT1, which is a protein-coding gene with 9831 base pairs located on chromosome 12p [28]. Whilst GD2 is expressed in normal brain tissues as well as in various neuroectodermal cancerous cells, it must be noted that NB is the only known solid tumor that expresses very high levels of the GD2 antigen, a characteristic feature that has been associated with a possible high copy number variation of B4GALNT1 [29]. Gene knockout experiments demonstrate gene-deficiency-related neurodegenerative disorders such as hereditary spastic paraplegia (HSP) [27], implying that careful consideration must be taken when developing a gene therapy that targets GD2 to avoid adverse effects. The expression characteristics of the GD2 synthase gene in cancerous cells or blood bone marrow can serve as a diagnostic tool to inform clinicians on treatment modalities. Overexpression of the human B4GALNT1 gene is also associated with adult T cell leukemia cell lines, glioma cell lines, some malignant melanomas, and breast cancer, and are detected by the presence and characteristic features of gangliosides in these cell lines [30,31]. This could partly explain why GD2-negative melanoma cells artificially induced to overexpress GD2 promote anchorage independence growth (an indication of tumorigenesis), cell migration in vitro, and enhanced tumor incidence in vivo [32].

Studies on the correlation between the GD2 gene and the ganglioside GD2 show that the GD2 synthase gene is directly responsible for the production of GD2 [33]. Blood serum and bone marrow are the main sites for the extraction of GD2 and GD2 genetic biomolecules, making the extraction process less intrusive [34,35]. Significantly high levels of free GD2 have been found in the serum of patients with NB compared with normal children and children with other tumors [36], indicating that NB ubiquitously expresses GD2. GD2 synthase mRNA has been derived from bone marrow (BM) and blood for use as a biomarker for NB [34]. However, it should be noted that although the specificity of the methods used to determine NB-mRNA (e.g., RTPCR assays) is high (detecting one tumor cell per 107 normal nucleated cells in as low as 100 pg of total RNA), it is not the most cost-effective and easily accessible method for the detection of NB MRD [37].

## 4. Minimal Residual Disease in NB

Minimal residual disease (MRD) is used as a prognostic indicator for risk-adapted therapy [38]. It generally involves the measurement of the amount of specific cells or biomolecular contents in the peripheral blood (PB) or bone marrow (BM) that are characteristics of a certain type of malignancy. More specifically, these molecules exist as drug-resistant tumor cells that appear as cancer stem cells (CSCs) in residual tumors, circulating tumor cells in PB, disseminated tumor cells in BM, and other metastatic sites [35]. MRD is the main cause of relapse in HR-NB. With a reputation of almost no recovery during this stage, the incidence is inevitable in late diagnosis and, therefore, patients will benefit greatly from timely, accurate, and easily available detection methods. There are several biomarkers available for the detection of NB-MR. These include CD133, CD114, CD57, and CD171 [39]. However, in our discussions, we limit the scope to currently available assays for detection of the GD2 antigen. We further discuss deep-learning-based methods that can be used to characterize MRD via GD2 expression.

## 5. GD2 as a Diagnostic Biomarker

### 5.1. Disialoganglioside (GD2)

**Figure 1:**
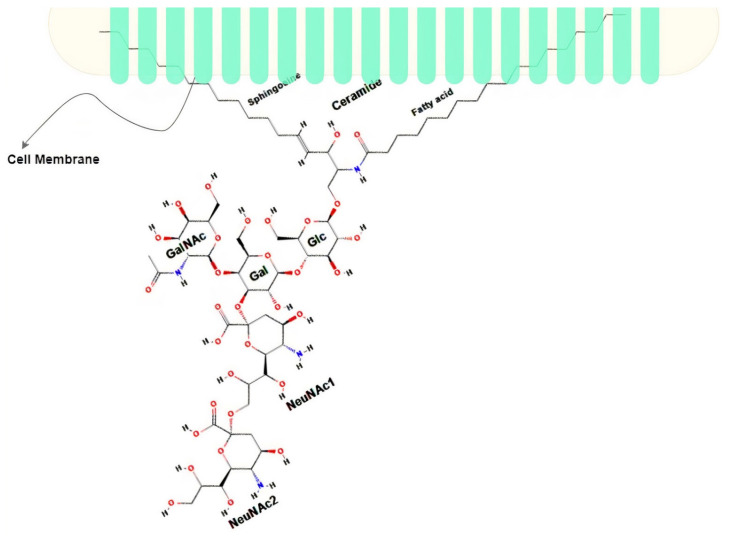
Structure of GD2 adapted from [26]; Sphingosine and Fatty acid are embedded in the cell membrane; Galactose (Gal), Glucose (Glc), N-acetylgalactosamine (GalNAc), and N-acetylneuraminic acid (NeuNAc) are the extracellular part.

Gangliosides are complex acidic glycosphingolipids containing at least one sialic acid (N-acetyl neuraminic acid or N-glycolylneuraminic acid) residue in their carbohydrate moiety. This characteristic translates into the existence of different variants of the gangliosides (about 188 variants as of 2004 [40]). Disialoganglioside (complex ganglioside) GD2 is a subtype of the ganglioside family that is normally associated with the plasma membrane of cells [41,42]. It comprises five monosaccharides linked to ceramide and embedded in the outer membrane with its ceramide tail while exposing its sugar moiety to the outer surface, as seen in Figure 1. It is known to be highly expressed during childhood development, initially being highly expressed and restrictive to the neural and mesenchymal stem cells during early development, and then finally restricted to the brain, peripheral neural fibers, and skin melanocytes [26,43]. Of all the neuroectodermally derived tumor cell lines and tissues studied by researchers, NB is the one known to have the highest expression of GD2 (about 98% expression in all NB cell lines) estimated at 5–10 million molecules per cell [26,44], making it an obvious biomarker for NB. GD2 is acknowledged as the 12th most potent antigen for cancer therapy [11]. Several immunotherapies have been developed to target GD2 with the most successful being Dinutuximab (a chimeric monoclonal antibody made from a combination of mouse and human DNA), which, when combined with immune stimulants such as interleukin-2 (IL-2), Granulocyte-macrophage colony-stimulating factor (GM-CSF), and cis-retinoic acid (CRA), demonstrates a higher event-free survival rate in patients with HR-NB compared with chemotherapy [44].

Although gangliosides in general play significant biological functions across many cell types (cell recognition and regulation of membrane-bound signaling proteins such as epidermal growth factor receptor (EGFR) and vascular endothelial growth factor receptor), the specific role played by GD2 in normal cells is not particularly known but thought to contribute to enhanced tumor cell proliferation, motility, migration, adhesion, invasion, and confers resistance to apoptosis in cancerous cells [26]. More research effort is needed to completely understand the structural and functional characteristics of GD2, including its binding characteristics, to facilitate the development of improved theranostics.

### 5.2. Neuroblastoma Detection: Focusing on GD2

Several assays presently exist for the detection of NB, especially in the blood and bone marrow. These include immunocytology, immunofluorescence, flow cytometry, and reverse transcriptasepolymerase chain reaction (RT-PCR) [35,45,46,47,48,49]. In this section, we will discuss how these conventional techniques are used for GD2 detection and describe novel approaches through the use of aptamers for the design of high-affinity molecules for GD2. The application of machine learning models for GD2 characterization based on high-throughput image processing and genomic analysis is also presented in this section.

### 5.3. NB Detection Techniques

Immunocytology and RT-PCR (biomarker-based assays) and imaging are presently the most sensitive detection techniques used in the diagnosis and characterization of NB in all stages of development. RT-PCR can detect one tumor cell in 106–107 hematopoietic cells and immunocytology is sensitive to the order of one tumor cell in about 104–105 [35,48,50]. RT-PCR amplifies the presence of genetic markers while immunocytology makes use of the presence of disease-specific antigens. Some genetic markers for NB include GD2 synthase mRNA, state of chromosome 1 (1p), MAGE and BAGE genes [46], and MYCN amplification; tumor-specific antigens include GD2, CD81, CD56, and CD45 [39].

As stated, immunocytology relies on the interaction between antibodies and tumor-associated cell surface or intracellular antigens that are specific to NB [35]. This method combines the principles of cytology with the specificity of immunological reactions to perform localization and/or quantification of cells with antigens of interest. In other words, the method thrives on the presence and development of antibodies or other high-affinity agents such as aptamers [51,52]. Although aptamers promise to overcome some of the limitations associated (in terms of selection difficulties, selectivity problems, preparation difficulties, high costs of production, stability, and cross-reactivity issues), there is little to no research reports on their application for GD2 characterization. Immunocytology detection characterizes histopathological features of malignant tissues or tumor cells present in bone marrow aspirate or biopsy [53]. By staining or radiolabeling either specific antibodies or aptamers that bind to malignant tumors or cells, it becomes possible to obtain enhanced images of the affected cell or tissue for further characterization.

RT-PCR is also effective for the detection of MRD [35] and mostly results in accurate depictions of the disease state. However, there remain, to date, issues related to false positives that are associated with DNA contamination of the target mRNA, making RT-PCR not the most optimal detection technique in all cases, even though it is highly sensitive [54,55]. These limitations present a challenge when detecting NB MRD because survivability is significantly connected with early, effective, and accurate diagnosis. Immunocytological assays with GD2 as the target antigen using highly specific bioaffinity ligands (e.g., aptamers) could improve the range of specificity compared with RT-PCR.

### 5.4. GD2 Detection with Monoclonal Antibodies

GD2 can be detected via the use of various radiolabeled antibodies, bispecific antibodies, and chimeric antigen receptor (CAR)-modified T cells [42,56] by utilizing various immunohistochemical staining techniques. Swerts et al., 2005 [57] is one of the premier reports to provide an internationally standardized protocol for the detection of NB MRD by immunocytochemistry using the monoclonal mouse antihuman GD2 disialoganglioside antibody. The authors provide two guidelines based on the morphological and immunocytological characteristics of GD2-positive cells, which serves as a means to reduce false-positives. A cell is GD2-positive if it has a round nucleus (larger than that of small lymphocytes), displays a granular chromatin structure, a scarce amount of cytoplasm, and displays a strong, deep, red stain localized to the entire cell membrane and cytoplasm. More than one antibody may be used to reduce the probability of false negatives [55]. Monoclonal antibodies (MAbs) that have been used to detect GD2-positive NB cell lines include MAb BW 625, UJ13A, UJ223-8, UJ127-11, UJ181 4, Thy-1, Hll ch14.18/CHO, Hu3F8, 131I-3F8 Hu14.18-IL-2, Hu14.18K322A, and ME36.1 [26,55,58]. It should be noted that although GD2-specific MAbs represent a promising future for GD2 detection, they are not as sensitive as RT-PCR. Aptamers have the potential to offer higher sensitivity, specificity, and better image quality due to their enhanced molecular binding stability [59].

### 5.5. GD2 Detection Using Aptamers

Aptamers are single-stranded oligonucleotides (RNA or DNA) capable of binding to specific target molecules [52,60]. Its application in the detection and treatment of several types of malignancy has been studied extensively with impressive results in binding specificities to various types of small molecules including antigens and toxins [61,62,63,64]. Beneficial properties of aptamers include their ability to be conjugated with other molecules to improve their stability and attach fluorescence molecule for imaging, to name a few [51,60,65,66]. In conjunction with flow cytometry, aptamers have been used to diagnose a myriad of tumors and other diseases targeting different cell surface antigens. Researchers have used in vivo Systematic Evolution of Ligands by Exponential Enrichment (SELEX) method to develop a novel DNA aptamer that is able to specifically bind GD2 antigens expressed by NB cells high affinity [67]. The method used by the researchers included not only e SELEX but also flow cytometry for experimental selection of DNA aptamer, and molecular docking for structure refinement followed by further synthesis of newly refined aptamers. Such an aptamer will be highly useful for diagnosis and staging of NB as well as detecting MRD. The development of such theranostics (e.g., aptasensors) for [68] improved histology will enable better cancer staging via increased contrast of the imaging obtained from tissue biopsies.

## 6. Computational Approaches for GD2 Characterization

### 6.1. Immunohistolocal Image Processing Pipeline

**Figure 2:**
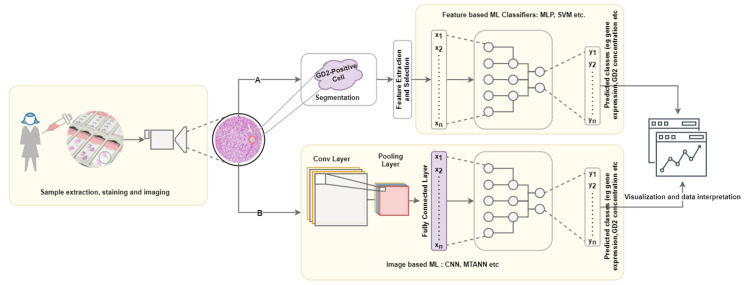
Pipeline for GD2-positive tissue/cell characterization; Path A represents feature-based ML, which is dependent on the effective of the chosen segmentation algorithm. Path B represents the image-based approach, which may only involve minimal preprocessing steps such as tiling. Image adapted from [93].

A typical computational pipeline used to process biomedical images to obtain meaningful insights is shown in Figure 2. It consists of four main steps: data acquisition and annotation, image prepossessing, machine learning application, data interpretation and visualization. This has further been grouped into three layers: wet lab, computational, and decision making with interconnections representing the iterations required to improve reliability.

In the wet-lab, tissue biopsies/cells for staining and imaging are collected using various tissue staining techniques. Staining utilizes the biology of cell-specific antigen/markers by using high-affinity molecules that bind specifically to the antigen of interest. Immunofluorescence, for instance, is a staining technique that has been applied to stain GD2 for the detection of NB using GD2-specific monoclonal antibody-like Ch14.18 [47,69] to generate very high-resolution whole slide images. Aptamers also serve as excellent molecular imaging probes for many cell- or tissue-imaging modalities due to their high target affinity and specificity, ease of synthesis compared to antibodies, fast tissue penetration ability, and thermal stability. Aptamer-mediated imaging has been explored by Shi et al. [70] using a Cy5-labeled aptamer named TD05 (Cy5-TD05) as the probe for imaging Ramos (B-cell lymphoma). Other researchers have also demonstrated the viability of aptamer-based imaging of various cancers using a variety of imaging techniques [71]. For instance, Hui et al. [72] developed an activatable aptamer probe (AAP) with the aptamer sgc8, which was selected via cell-SELEX against human acute lymphoblastic leukemia CCRFCEM cells for in vivo cancer imaging. The AAP was a single-stranded oligonucleotide consisting of three cojoined fragments: the aptamer sequence, a poly-T linker, and a short DNA sequence with a fluorophore and a quencher covalently attached at either terminus. This arrangement of fragments displayed enhanced imaging contrast when compared to probes with nonswitching mechanisms during in vivo testing. Additionally, aptamer-mediated imaging of cancer cells has been utilized in Magnetic Resonance Imaging (MRI) [73,74,75,76], Single-Photon Emission Computed Tomography [77], Positron-Emission Tomography [78,79], Computed Tomography [80,81], and Ultrasound [82], thus showing the potential of aptamers in several tissue or cell imaging protocols. The development of GD2 aptamers, as reported by Zhang et al. [67], will contribute greatly to the characterization and detection of GD2 via different cell/tissue-imaging techniques. Processing the huge amount of information that is generated through these aptamer-mediated imaging techniques requires the use of computational approaches.

The computational layer may involve image labeling, which is simply the annotations or labels associated with the image feature vectors. For GD2 characterization, feature vectors may be mapped to GD2 concentrations from detection assays, RNA-sequence data, and/or other quantifiable annotations derived from both algorithmic sources and human knowledge. As an example, Schmauch et al., 2020 [83] used RNA-seq data as labels for histological images from the Cancer Genomic Atlasto to build transcriptome prediction models. Another important step in this layer is the preprocessing step, which may involve tiling of whole slide images (WSI) due to the high dimensionality and small sample sizes of the WSI [83,84], image augmentation and/or feature vector extraction using an encoder–decoder architecture, and other feature extraction algorithms or Principal Components Analysis (PCA) for dimensionality reduction.

The success of the last step of building the model largely depends on that of the previous steps. Some appropriate Machine learning (ML) models include classification, regression, and/or segmentation models [85] such as VGGNet [86], ResNet [87], Inception [88], UNET [89], FCN [90], FastSCNN [91], and DeepLab [92]. An extensive discussion of various deep learning models of digital histopathology has been reported previously [85].

### 6.2. Feature Extraction

Feature extraction is a method used to represent the state of an image as a simple vector where each vector component defines a specific measurable attribute of the image. The main aim of feature extraction for GD2-stained tissues biopsies or cell culture WSIs is to provide a means for quantifying stained cells with the potential to map their quantities to disease state including, but not limited to stage [94,95,96,97], gene expression profile [98], GD2 concentration, etc. Due to the high dimensionality of the image data (typically 100,000 × 100,000 pixels [99,100]), performing feature extraction is a necessary step for many biological image processing techniques as these features could correspond directly to disease state.

Possible features worth extracting can be categorized into four main groups; morphometry, intensity, texture, and gradient statistics [101]. Liu et al., 2016 [102] for instance, using a supervised learning approach, performed nuclear segmentation of follicular adenoma, nodular goiter, and follicular variant papillary carcinoma stained images using the semiautomatic segmentation model developed by Chen et al., 2013 [103] and further represented the features of each nucleus with a 256-dimensional numerical vector, which included 6 morphological features (area, convexity, circularity, perimeter, eccentricity, and equivalent diameter), 220 texture features (intensity-based features, Haralick features, and Gabor features), and 30 wavelet features. This was then followed by PCA to extract the top 20 features for each nucleus to be used as the final feature vector for classification by a support vector machine (SVM) architecture.

Although feature extraction from segmented cells presents a great opportunity to characterize GD2-positive cells in NB MRD, the reliance on segmentation of cells may be challenging, especially for complicated structures with complex backgrounds. A deep learning (whole-image-based ML) method, which provides an end-to-end pipeline that overcomes the problem of inaccurate feature calculation and segmentation, can be used for direct mapping of GD2-stained WSI to labels of interest.

## 7. Conclusions

The ganglioside GD2 is expressed abundantly in NB and, therefore, presents great opportunities to develop novel theranostics tools for NB. With the development of GD2-specific monoclonal antibodies as well as the development of high-affinity GD2-aptamers for immunohistochemistry and the advancement of machine learning methods for high-throughput imaging analysis, it is possible to develop methods for quantifying and characterizing GD2 expression levels in a diseased cell or tissue.

Furthermore, this approach can be used for subclinical disease monitoring and targeting of NB at the molecular level towards the development of advanced theranostics for NB treatment as well as facilitating treatment augmentation to reduce the occurrence of relapse. The approach can also be extended to different types of cancers, tumors, and other diseases with unique, cell-mediated receptors or biomarking phenomena to develop novel theranostics for diagnosis and treatment. Applications of computational approaches such as machine (deep) learning algorithms, as discussed in the article, can be used to decipher diseases with similar signaling molecules and characteristics using the expression features of the signaling molecules and novel bioaffinity systems to facilitate the development of high-precision biomedical tools for preclinical and clinical applications.

This article briefly discussed the structural and functional features of GD2, the emergence of aptamers for high-affinity GD2 targeting, and the application of machine learning approaches for the detection and quantification of GD2 for MRD characterization via high-throughput image processing.
